# Abnormal Spontaneous Neural Activity of the Central Auditory System Changes the Functional Connectivity in the Tinnitus Brain: A Resting-State Functional MRI Study

**DOI:** 10.3389/fnins.2019.01314

**Published:** 2019-12-20

**Authors:** Wei-Wei Cai, Zhi-cheng Li, Qin-tai Yang, Tao Zhang

**Affiliations:** ^1^Department of Otolaryngology–Head and Neck Surgery, The First Affiliated Hospital of Jinan University, Guangzhou, China; ^2^Department of Otolaryngology–Head and Neck Surgery, Panyu Central Hospital, Guangzhou, China; ^3^Department of Otolaryngology–Head and Neck Surgery, The Third Affiliated Hospital, Sun Yat-sen University, Guangzhou, China

**Keywords:** tinnitus, resting-state functional magnetic resonance imaging, amplitude of low-frequency fluctuation, auditory cortex, auditory pathway, spontaneous neural synchrony

## Abstract

**Objective:**

An abnormal state of the central auditory system (CAS) likely plays a large role in the occurrence of phantom sound of tinnitus. Various tinnitus studies using resting-state functional MRI (RS-fMRI) have reported aberrant spontaneous brain activity in the non-auditory system and altered functional connectivity between the CAS and non-auditory system. This study aimed to investigate abnormal functional connections between the aberrant spontaneous activity in the CAS and the whole brain in tinnitus patients, compared to healthy controls (HC) using RS-fMRI.

**Materials and Methods:**

RS-fMRI from 16 right-ear tinnitus patients with normal hearing (TNHs) and 15 HC individuals was collected, and the time series were extracted from different clusters of a CAS template, supplied by the Anatomy Toolbox of the Statistical Parametric Mapping software. These data were used to derive the smoothed mean amplitude of low-frequency fluctuation (smALFF) values and calculate the relationship between such values and the corresponding clinical data. In addition, clusters in the CAS identified by the smALFF maps were set as seed regions for calculating and comparing the brain-wide connectivity between TNH and HC.

**Results:**

We identified the different clusters located in the left higher auditory cortex (HAC) and the right inferior colliculus (IC) from the smALFF maps that contained increased (HAC) and decreased (IC) activity when the TNH group was compared to the HC group, respectively. The value of increased smALFF cluster in the HAC was positively correlated with the tinnitus score, but the decreased smALFF cluster in the IC was not correlated with any clinical characters of tinnitus. The TNH group displayed increased connectivity, compared to the HC group, in brain regions that encompassed the left IC, bilateral Heschl gyrus, bilateral supplementary motor area, right insula, bilateral superior temporal gyrus, right middle temporal gyrus, left hippocampus, left amygdala, and right supramarginal gyrus.

**Conclusion:**

Tinnitus may be linked to abnormal spontaneous activity in the HAC, which can arise from the neural plasticity induced from the increased functional connectivity between the auditory network, cerebellum, and limbic system.

## Introduction

Tinnitus is commonly defined as the perception of sound in the absence of an external auditory source and has a prevalence ranging from 10 to 15% in the adult population (Xu et al., 2011; Heller, 2003). Many patients with tinnitus have reported symptoms that can significantly impair patients’ quality of life (Langguth, 2011), such as frustration, annoyance, irritability, anxiety, depression, hearing difficulties, hyperacusis, insomnia, and concentration difficulties.

Most tinnitus accompanies obvious hearing loss, but some patients without hearing loss can also experience severe tinnitus (Savastano, 2008; Martines et al., 2010). Evidence from studies using animal models of tinnitus indicates that long-term exposure to non-traumatic noise can (Munguia et al., 2013; Lau et al., 2015; Takacs et al., 2017) induce dysfunctional brainstem projections from the cochlea that can trigger the development of abnormal neuronal baseline activity in the several levels of central auditory system (CAS).

NeuroImage techniques applying to tinnitus human studies include testing abnormally elevated spontaneous activity, investigations of brain structure, and exploring corresponding changes in non-auditory system (NAS) following tinnitus perception (Landgrebe et al., 2009; Chen et al., 2014; Geven et al., 2014; Seydell-Greenwald et al., 2014). Tinnitus is the perception of phantom sound in the absence of physical signal and is no task-based modulation of the tinnitus signal. For these reasons, tinnitus may be uniquely suited to studies using resting-state functional MRI (RS-fMRI).

Functional brain regions such as motor cortices (Biswal et al., 1995), visual cortices (Kiviniemi et al., 2004), auditory cortices (Cordes et al., 2001), and default mode network (Fox et al., 2005) that have been identified using stimulus- or task-evoked paradigms can also be identified in the resting state by examining low-frequency (0.01–0.08 Hz) fluctuations (LFFs) of the resting-state blood oxygen level dependent (BOLD) signal. These results imply that the LFFs infer neuronal activation indirectly through neurovascular coupling (Lu et al., 2007; Mantini et al., 2007; Shmuel and Leopold, 2008; Rusiniak et al., 2015; Shtark et al., 2015). These networks generally show reliable and consistent patterns of functional connectivity (FC) (Zhang et al., 2008), which is defined as the quantification of the operational interactions of multiple spatially distinct brain regions that are highly synchronous (Rogers et al., 2007). The effects of tinnitus on resting-state FC have recently been reported by using RS-fMRI and have variable results for experimental and analytical methods, partly due to heterogeneity of tinnitus subjects (Burton et al., 2012; Kim et al., 2012; Maudoux et al., 2012a, b; Wineland et al., 2012; Davies et al., 2014). These studies obtained the modulated correlation between the auditory resting-state network and the following networks: default mode, limbic, dorsal attention, and visual networks to explain the mechanism of tinnitus accompany symptoms.

The amplitude of LFFs (ALFFs), an alternative index to LFF, has been suggested as a measure of regional spontaneous neuronal activity and has been validated using a comparison between ALFF and PET imaging (Zang et al., 2007; Yuan et al., 2018). The ALFF metric has previously been used to measure intrinsic regional neural activity at the baseline state of neurological or psychiatric disorder such as attention deficit hyperactivity disorder, Parkinson disease, and depression (Rogers et al., 2007; Zang et al., 2007; Zhang et al., 2008; Skidmore et al., 2013; Huang et al., 2017; Lei et al., 2017; Li et al., 2017). In a recent tinnitus study, the ALFF has been used to reveal abnormal spontaneous neural activity in tinnitus patients (Chen et al., 2014; Lv et al., 2016) and found that aberrant ALFF brain regions (right middle temporal gyrus) in the NAS are associated with clinical tinnitus characteristics (Chen et al., 2014). However, changes in the regional spontaneous neural activity of the CAS are thought to be the first stage of sound perception in tinnitus. In fact, these changes may be the driving factor for aberrant global FC, which can induce the various disease symptoms except for phantom sound. Therefore, the mechanism of altered FC of tinnitus needs to be explored further to understand the relationship between the abnormal neural activity within the CAS and the NAS regions.

Previous RS-fMRI studies have explored aberrant FC between the CAS and the NAS to interpret various clinical dysfunctions such as anxiety and depression, which often accompany tinnitus (Burton et al., 2012; Maudoux et al., 2012a, b; Wineland et al., 2012; Davies et al., 2014). The CAS in some of these studies was isolated from the rest of the brain using independent component analyses to explore the relationship between the whole CAS and NAS (Kim et al., 2012; Maudoux et al., 2012b; Davies et al., 2014). Other studies identified the auditory cortex based on a human brain atlas and used such regions as seeds to assess abnormal FC across the whole brain (Burton et al., 2012; Wineland et al., 2012). These methods selected the whole CAS or primary auditory cortex (PAC) regardless of its active vs. inactive regions caused by tinnitus perception. While proven to be useful, the assumptions of these techniques in the processing pipeline might lead to some inaccuracies in the resulting connectivity measures and the subsequent clinical implications.

Base on prior work and theoretical consideration, we try to improve the consistency of tinnitus subjects including several factors such as unilateral tinnitus ear, the possible causes of tinnitus, and handedness to make the differences prominent between groups. The present study attempted to assess abnormal activity of the tinnitus brain and the functional connections thereof using active clusters within the CAS that were identified through a data-driven approach utilizing the ALFF metric. We hypothesized that tinnitus patients exhibit abnormal FC between various clusters of the CAS and the remainder of the brain.

## Materials and Methods

### Subject Demographics

This study utilized data from two groups of subjects, tinnitus patients with normal hearing (TNHs) and healthy controls (HCs), who were recruited from various sources. The TNH group was composed of subjects recruited from outpatient clinics at The Otorhinolaryngology Department of Guangzhou Panyu Central Hospital, The First Affiliated Hospital of Jinan University, and The Third Affiliated Hospital, Sun Yat-sen University, Guangzhou, Guangdong, China, from March 2015 to May 2018. The HC group was matched in age, sex, and education to the TNH group. The TNH group comprised 16 right-handed patients between the ages of 18 and 55 years with self-reported right-ear tinnitus lasting >6 months. All tinnitus patients have occupational noise exposure or have experienced environmental noise exposure. The HC group contained 15 subjects who were recruited from the staff of Jinan University. All individuals provided written informed consent before participation in this study. This study was approved by the Research Ethics Committee of Guangzhou Panyu Central Hospital and the First Affiliated Hospital of Jinan University, Guangzhou.

**FIGURE 1:**
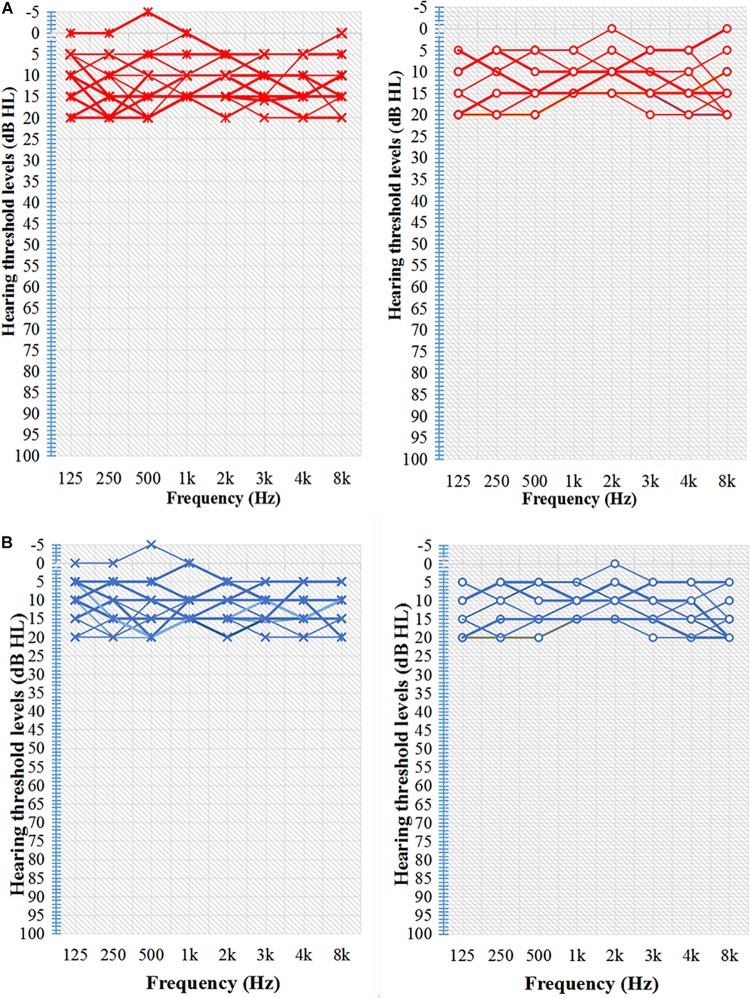
Characteristic hearing levels of the groups. **(A)** TNH group hearing threshold (*n* = 16). **(B)** HC group hearing threshold (*n* = 15). X, left ear air conduction; O, right ear air conduction; TNH, tinnitus patients with normal hearing; HC, healthy controls. Red, hearing threshold audiogram of TNH; Blue, hearing threshold audiogram of HC.

Pure-tone audiometry was performed with a clinical audiometer using eight octave frequencies (0.125, 0.25, 0.5, 1, 2, 3, 4, and 8 kHz) in both the TNH and HC groups to determine audio thresholds. The audio thresholds of the TNH patients and HC are shown in Figure 1 and Table 1. The severity of tinnitus was assessed by a Chinese translation of the Tinnitus Handicap Inventory (THI), a self-reported tinnitus handicap questionnaire (Meng et al., 2012). All subjects were confirmed to have a Self-Rating Depression Scale (SDS) score of <50 and a Self-Rating Anxiety Scale score of <50 (Zung, 1971, 1986).

**TABLE 1:** Characteristic hearing levels of the participants.

	**125 Hz**	**250 Hz**	**500 Hz**	**1 kHz**	**2 kHz**	**3 kHz**	**4 kHz**	**8 kHz**
TNH (right ear, *n* = 16)	15.00 ± 5.477	13.75 ± 5.916	13.44 ± 5.692	11.25 ± 3.416	10.31 ± 4.270	12.50 ± 4.082	13.44 ± 4.732	14.06 ± 5.836
HC (right ear, *n* = 15)	15.00 ± 5.669	14.00 ± 6.036	13.33 ± 5.876	12.00 ± 3.519	10.33 ± 4.419	12.67 ± 4.169	13.67 ± 4.806	14.33 ± 5.300
*P*-value (*df* = 29)	1.000	0.908	0.960	0.947	0.989	0.911	0.895	0.894
TNH (left ear, *n* = 16)	10.63 ± 5.737	11.25 ± 6.191	11.56 ± 5.876	10.63 ± 5.123	10.31 ± 4.990	11.94 ± 4.090	12.19 ± 4.820	11.88 ± 5.737
HC (left ear, *n* = 15)	10.33 ± 5.815	11.33 ± 6.399	12.00 ± 7.270	11.00 ± 5.071	10.67 ± 4.952	12.07 ± 4.200	12.33 ± 4.952	12.00 ± 5.278
± *P*-value (*df* = 29)	0.889	0.971	0.868	0.839	0.844	0.931	0.934	0.950

*TNH, tinnitus patients with normal hearing; HC, healthy controls. The auditory threshold data of TNH and HC are represented as mean ± SD.*

We excluded subjects who exhibited any of the following criteria: Mènière disease; conductive deafness; alternative hearing level; cognitive or mental disorders; serious systemic diseases, such as heart failure or diabetes; epilepsy; alcoholism or use of psychiatric drugs; pregnancy; acoustic neuroma, brain stem, or inferior colliculi diseases; hyperacousia; smoking; and history of stroke, brain injury, Alzheimer’s disease, or Parkinson’s disease. Table 2 summarizes the characteristics of the TNH and HC subjects in this study.

**TABLE 2:** Clinical characteristics of study subjects.

	**TNH**	**HC**	***P*-value**
Gender (male/female)	6/10	5/10	0.689
Age (years)	35.33 ± 10.70	35.00 ± 10.10	0.720
Education (years)	13.50 ± 1.57	13.25 ± 1.75	0.698
SDS score	34.67 ± 7.30	35.25 ± 6.33	0.274
SAS score	35.67 ± 9.37	37.65 ± 7.23	0.240
THI score	55.33 ± 11.03	\	\
Tinnitus duration (months)	36.58 ± 18.03	\	\
Frequency range of tinnitus	5–8 kHz	\	\

*SDS, Self-Rating Depression Scale; SAS, Self-Rating Anxiety Scale; THI, Tinnitus Handicap Inventory.*

### MRI Data Acquisition

Resting-state BOLD fMRI data were acquired with a 3.0 T MR scanner (MR750, GE Health Care System, United States) in the Imaging Center of the First Affiliated Hospital of Jinan University. Before the MRI, all subjects laid in the supine position on the examination bed with noise-cancelling headphones and earplugs (Mack’s noise reduction ear plugs, United States) to reduce the noise level by 32 dB. To reduce head motion, a foam pad was fixed around the subjects’ head for the duration of the scan. All subjects were asked to remain relaxed with their eyes closed and to avoid serious thought for approximately 20 min. In order to avoid the influence of acoustic noise produced during the MRI procedure on brain activity, the BOLD images were acquired using an echo planar-imaging sequence with the following parameters: repetition time (TR) = 2000 ms, echo time (TE) = 30 ms, slice thickness = 3 mm, flip angle = 90°, field of view = 200 × 200 mm, acquisition matrix = 64 × 64 mm, and the number of time points (volumes) = 160. Anatomical images were acquired using a 3D-fast spoiled gradient echo sequence with the following parameters: TR = 7.63 ms, TE = 3.74 ms, slice thickness = 1 mm, flip angle = 8°, field of view = 256 × 256 mm, and acquisition matrix = 256 × 256 mm. The MRI images included the whole brain.

### Data Processing

All fMRI data were processed using the DPAESF (Data Processing Assistant for Resting-state fMRI) toolbox (Chao-Gan and Yu-Feng, 2010), which is embedded in the DPABI (Data Processing and Analysis for Brain Imaging) V4.3 toolbox (Yan et al., 2016), and based on Statistical Parametric Mapping SPM^1^. First, the initial 10 volumes were discarded, and slice-timing correction was performed, with all volume slices being corrected for different signal acquisition time by shifting the signal measured in each slice relative to the acquisition of the slice at the mid-point of each TR. Then, the time series of images for each subject were realigned using a six-parameter (rigid body) linear transformation with a two-pass procedure (registered to the first image and then registered to the mean of the images after the first re-alignment). After realignment, individual T1-weighted map were co-registered to the mean functional image using a 6 degree-of-freedom linear transformation without re-sampling and then segmented into gray matter, white matter, and cerebrospinal fluid (Ashburner and Friston, 2005). Finally, the transformations from individual native space to Montreal Neurological Institute (MNI) space were computed with the Diffeomorphic Anatomical Registration Through Exponentiated Lie algebra (DARTEL) tool (Ashburner, 2007). To control for head motion confounds, we utilized the Friston 24-parameter model (Friston et al., 1996) to regress out head motion effects. The Friston 24-parameter model (i.e., 6 head motion parameters, 6 head motion parameters one time point before, and the 12 corresponding squared items) was chosen based on prior work that higher-order models remove head motion effects better (Yan et al., 2013). In addition, mean FD was used to address the residual effects of motion in group analyses. Mean FD is derived from Jenkinson’s relative root mean square algorithm. We used the threshold (mean FD Jenkinson, <0.2 mm) to exclude the subjects, which followed the research about the impact of head motion on RS-fMRI (Jenkinson et al., 2002). The mean FD_Jenkinson values of subjects in this study were max = 0.13, min = 0.03, and mean = 0.05 SD. None of the subjects were excluded. Global signal regression and sources of spurious variance (white matter and cerebrospinal fluid signals) were also removed from the data through linear regression to reduce respiratory and cardiac effects. In addition, linear trends were included as a regressor to account for drifts in the BOLD signal. We performed temporal filtering (0.01–0.1 Hz) on all time series except for ALFF. The ALFF results were then standardized by global mean to generate mALFF maps. Then, mALFF maps were smoothed with a Gaussian kernel [FWHM = (8 × 8 × 8) mm] to generate smoothed mean amplitude of low-frequency fluctuation (smALFF) maps. A larger smoothing kernel (8 mm) was used in this work as it has been shown to improve the reproducibility of results and more likely reflects the true effect for group comparison (Mikl et al., 2008).

### CAS Template Setting

**FIGURE 2:**
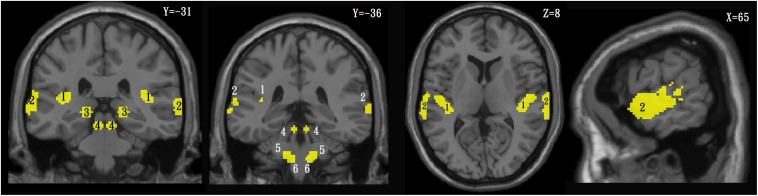
Template of the central auditory system in the coronal (left), axial (middle), and sagittal (right) planes. 1 = primary auditory cortex, 2 = higher auditory cortex, 3 = medial geniculate nucleus, 4 = inferior colliculus, 5 = superior olivary complex, 6 = cochlear nuclei.

In order to identify the different CAS active clusters between the TNH and HC groups, the SPM anatomy toolbox v1.8^2^ was used to create a template composed of the PAC and the combination of areas Te 1.0, Te1.1 (Brodmann area 41), Te1.2 (Brodmann area 42) the higher auditory cortex (HAC), and area Te3.0 (part of Brodmann area 22 and Brodmann area 42) (Morosan et al., 2001, 2005). The auditory pathway template was based on regions of interest (ROIs) reported by Muhlau et al. (2006). The ROIs are shown in Figure 2 and were defined as the dorsal cochlear nuclei (sphere radius: 5 mm; MNI coordinates, ± 10, −38, −45), superior olivary complex (sphere radius: 5 mm; MNI coordinates, ±13, −35, −41), inferior colliculus (IC; sphere radius: 5 mm; MNI coordinates, ±6, −33, −11), and medial geniculate nucleus (sphere radius: 8 mm; MNI coordinates, ±17, −24, −2) (Muhlau et al., 2006). The SPM Marsbar toolbox was employed for ROI analysis of the HAC, PAC, and auditory pathway using the CAS template (Brett et al., 2002; Figure 2).

### Statistical Analysis

The statistical analysis tool embedded within DPABI was used to calculate the difference between the THN and HC groups using two-sample *t*-tests. A permutation test was used to correct for multiple comparisons (Winkler et al., 2016), which offered the optimal balance between the family-wise error rate and test-retest reliability (Chen et al., 2018). The permutation test was two-tailed and performed with a threshold controlling family-wise error rate at *p* < 0.05 level, 5000 permutations, and no acceleration.

Comparing the smALLF maps of the TNH with HC groups within the CAS template was performed using two-sample *t*-tests, and the gray matter images of the TNH and HC groups were added as a covariate. To investigate the relationship between the neural activity and the clinical data of tinnitus patients, we extracted smALFF values from different clusters in smALFF maps of the TNH group and computed the Pearson’s correlation coefficient with each clinical characteristic available (Table 2).

The different clusters of smALFF were defined as seeds to explore the different FC patterns (comparing TNH and HC); the time series of the seed voxels were used to calculate the Person correlation coefficient across the whole brain, which was defined by Whole-Brain-template of SPM8, and Whole-brain-template covers the CAS template. The FC maps using different smALLF clusters as seed regions were compared between the TNH and HC groups using two-sample *t*-tests.

In order to analyze the asymmetries of tinnitus brain in CAS, smALFF values of the left/right PAC and HAC were extracted from smALFF maps of all subjects to compare differences between the left and right auditory cortex in group and calculated the left/right smALFF ratio to compare difference between groups. An independent samples *t*-test examines the statistic differences in group and between groups. Statistical analysis was carried out using SPSS 18.0 package for Windows.

## Results

### Comparison of smALFF in the CAS Between Groups

**FIGURE 3:**
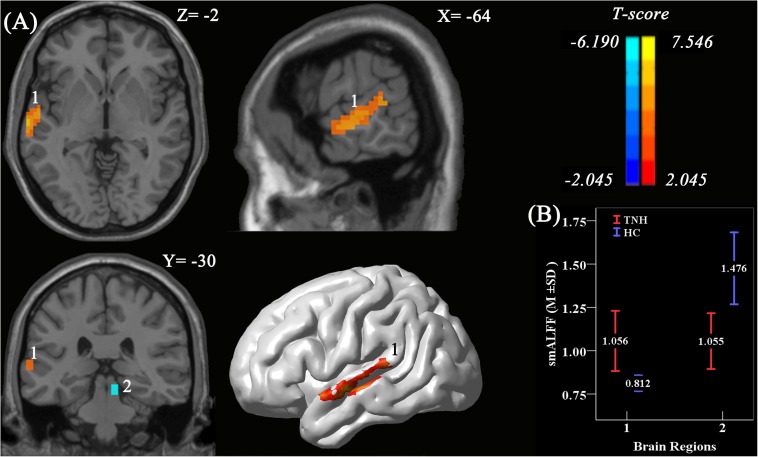
Significant smoothed mean amplitude of low-frequency fluctuation (smALFF) differences in tinnitus patients without hearing loss, compared to healthy controls. **(A)** The differences maps at slices *Z* = –2, *X* = –64, *Y* = –3,0 and a 3D rendering of the left-lateral view of the brain. The permutation test was two-tailed and performed with a threshold controlling family-wise error rate at *p* < 0.05 level, 5000 permutations, and no acceleration. Blue indicates that TNH subjects had decreased smALFF compared to the HC, and the yellow indicates the opposite. Number 1 indicates that the cluster of increased smALFF value located in the left higher auditory cortex (HAC; *T* value’s peak score: 7.55; MNI: –57, –6, –9; cluster size: 142 voxels), and number 2 indicates the cluster of decreased smALFF value located in the right inferior colliculus (*T* value’s peak score: –6.19; MNI: 9, –30, –15; cluster size: 16 voxels). **(B)** The smoothed mean and SD of standardized ALFF at the peak voxels.

Analyses using two-sample *t*-tests revealed clusters with significantly different smALFF values between the TNH and HC groups. A significantly increased smALFF cluster was found in the left HAC, and a significantly decreased smALFF cluster was found in the right IC (Figure 3). The cluster in the HAC was 142 voxels large, and had a peak intensity of *t* score = 7.55 and an MNI location of peak intensity of (−57, −6, −9). The peak intensity of the active cluster was located in the left middle temporal gyrus (Automated Anatomical Labeling atlas) or Brodmann area 22.

**FIGURE 4:**
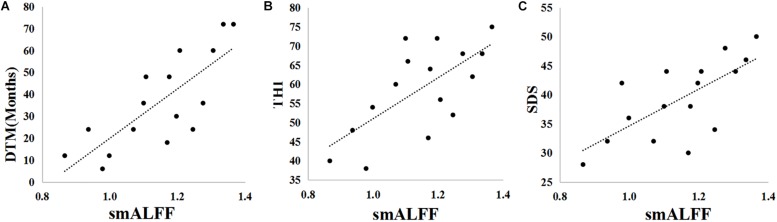
Pearson correlations between clinical parameters [Duration of Tinnitus-Months (DTM), Tinnitus Handicap Inventory (THI) score, Self-Rating Depression Scale (SDS) score, and Self-Rating Anxiety Scale (SAS) score] and the smALFF values of the active cluster in the left HAC. **(A)** DTM-smALFF: *r* = 0.778, *p* = 0.000. **(B)** THI-smALFF: *r* = 0.682, *p* = 0.004. **(C)** SDS-smALFF: *r* = 0.694, *p* = 0.003.

In the TNH group, the average smALFF value of the different cluster of left HAC cluster was correlated with various clinical metrics, including the Duration of Tinnitus-Months (DTM) (*P* < 0.01), THI (*P* < 0.01), and SDS (*P* < 0.01) (Figure 4).

### Brain-Wide Functional Connectivity With the CAS

**FIGURE 5:**
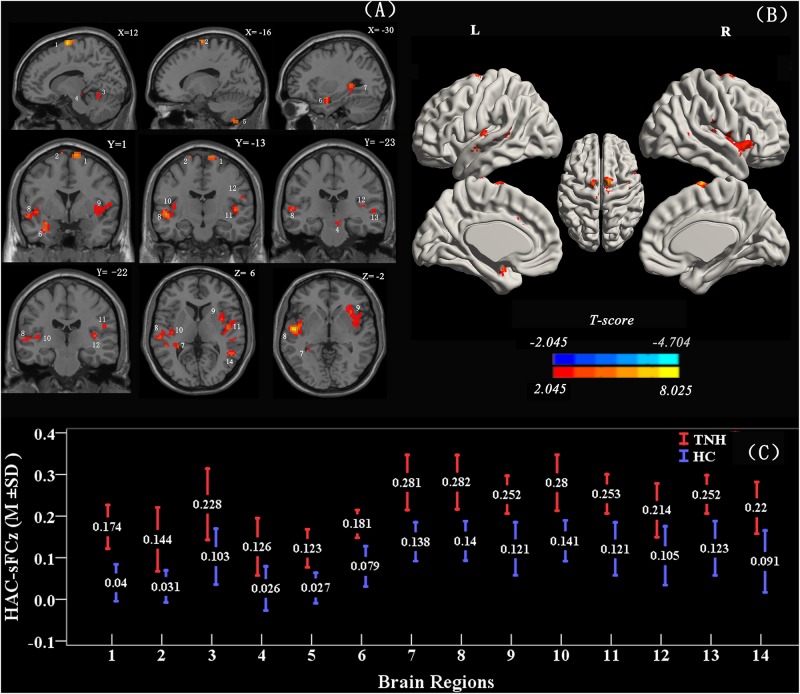
Clusters displaying a significant difference in functional connectivity (FC) (smoothed-Z-transfer maps) between the TNH and HC groups. Results are presented as slices **(A)** and a 3D rendering **(B)**. The HAC cluster from Figure 3 was used as the seed region to explore the functional connectivity across the whole brain. The permutation test was two-tailed and performed with a threshold controlling family-wise error rate at *p* < 0.05 level, 5000 permutations, and no acceleration. Blue indicates that TNH subjects had decreased smoothed-Z-transfer FC compared to the HC, and the yellow indicates the opposite. **(C)** The smoothed-Z transformed standardized functional connectivity at the peak voxels. Numbers (1–14) indicate the different clusters; see Table 3 for the corresponding brain areas and the cluster characteristics.

When using the left HAC active cluster as the seed region for investigating brain-wide FC, we found differences in this global connectome between the TNH with HC groups (Figure 5). The TNH group revealed significantly increased FC between the HAC and the bilateral supplementary motor area, bilateral insula, bilateral superior temporal gyrus (STG), bilateral Heschl, left hippocampus, left amygdala, left IC, left rolandic operculum, left cerebellum (lobule 8), and right cerebellum (lobules 4, 5) (see Figure 5 and Table 3). We found no regions that displayed a significant decrease in FC in the TNH group compared to the HC group. When using the right IC cluster as the seed, no active clusters survived the FC comparison between the TNH and HC groups.

**TABLE 3:** Characteristics of clusters displaying significantly different functional connectivity between the TNH and HC groups.

**Number order of the clusters**	**Brain regions (AAL)**	**Peak intensity (T-score) (L/R)**	**Peak MNI coordinate**	**Number of voxels**	**Function of brain region**
10, 11	Heschl (L/R)	4.56/5.23	−38, −19, 5/45, −13, 5	14/19	Auditory network
8, 13	Superior temporal gyrus (L/R)	7.50/4.51	−51, −9, −6/51, −37, 12	130/34	Auditory network
14	Middle temporal gyrus (R)	5.13	51, −51 6	28	Auditory network
4	Inferior colliculus (R)	4.16	3, −27, −15	11	Auditory network
3	Cerebellum 4,5 (L)	4.75	15, −57, −18	24	Motor planning and control
5	Cerebellum 8 (L)	6.48	−18, −63, −60	20	Motor planning and control
7	Hippocampus (L)	5.47	−30, −42, 3	55	Mood, memory, spatial navigation
6	Amygdala (L)	5.50	−30, 0, −18	27	Emotions; fear or anxiety
13	Supramarginal gyrus (R)	4.54	55, −21, 26	14	Dorsal attention network
9	Insula (R)	5.73	45, −9, 3	148	Executive control of attention
1, 2	Supplementary motor area (L/R)	6.10/8.02	−15, −6, 75/9, −3, 75	18/53	Motor control

*AAL, Automated Anatomical Labeling atlas; MNI, Montreal Neurological Institute; R, right; L, left.*

### Asymmetric Analysis of Auditory Cortex With smALFF in Group and Between Groups

Table 4 shows the characters of value smALFF extraction from bilaterality PAC and HAC, respectively. An independent samples *t*-test examines the statistic differences in group and between groups. Statistical analysis was carried out using SPSS 18.0 package for Windows. The asymmetric analysis results were as follows: (1) the HC group shows a higher smALFF value on the left PAC (*P* = 0.048) and right HAC (*P* = 0.001); (2) the TNH group shows no significant difference between the bilateral auditory cortex; and (3) when the asymmetry ratio (left/right auditory cortex’s smALFF value) between TNH and HC was compared, there is no significant difference between TNH and HC: *P* (HAC) = 0.059 and *P* (PAC) = 0.993.

**TABLE 4:** Asymmetric analysis of the auditory cortex in group and between groups (analysis with smALFF).

				***P*-value**
**Items**	**Side**	**TNH (*n* = 16)**	**HC (*n* = 15)**	**(TNH/NC)**
PAC	Left	1.136 ± 0.267	1.041 ± 0.073	
PAC	Right	1.079 ± 0.161	0.990 ± 0.061	
*P-*value (L/R)		0.472	0.048	
HAC	Left	0.844 ± 0.120	0.707 ± 0.046	
HAC	Right	0.952 ± 0.214	0.871 ± 0.044	
*P-*value (L/R)		0.093	0.001	
HAC ratio (L/R)		0.926 ± 0.214	0.813 ± 0.066	0.059
PAC ratio (L/R)		1.053 ± 0.170	1.053 ± 0.069	0.993

*smALFF, smoothed mean amplitude of low-frequency fluctuation; PAC, primary auditory cortex; HAC, higher auditory cortex.*

## Discussion

In the present study, we hypothesized that tinnitus may be the result of alterations in the spontaneous activity of the brain, and that changes in resting state CAS activity cause aberrant brain-wide FC that leads to the stress of tinnitus through plastic changes of neural behavior.

To improve the consistency of tinnitus group for making different activity in brain prominent between groups, it have been concerned several influenced factors including unilateral tinnitus, hearing level of tinnitus ear, right handedness, and the cause of tinnitus for the following reasons. (1) right ear tinnitus might have a different THI score when compared with left ear tinnitus, with the right ear having an advantage for speech sounds, and peripheral and cognitive interaction (Tai and Husain, 2018); (2) unilateral tinnitus and right handedness might help in increasing a significant difference in spontaneous activity in the tinnitus brains for the possible link between tinnitus and hemisphere dominance (Reiss and Reiss, 2001); (3) the unified cause of tinnitus may have similar mechanism. The non-traumatic noise-induced tinnitus has been found regional spontaneous neural activity in the auditory cortex and pathway (Schaette and McAlpine, 2011; Munguia et al., 2013).

In CAS, we found that smALFF maps of TNH showed hyperactivity in the left HAC (BA22) and reduced activity in the right IC (Figure 3) when compared with HC.

It is not sure if the increasing smALFF cluster of HAC might reflect the abnormal asymmetry or regional spontaneous neural activity of the auditory cortex caused by tinnitus. We analyzed the asymmetry of every subject with smALFF value extracted from the PAC and HAC. We found that the asymmetric feature is present in HC but not in TNH, and there is no significantly different asymmetric ratio between TNH and HC (Table 4). Geven et al. have a similar finding on asymmetric feature in the tinnitus and control groups (Geven et al., 2014). In previous tinnitus studies on baseline activity measurements using PET, Arnold et al. (1996) found that the left PAC had significantly more asymmetric activity in tinnitus patients and the control group also showed the same asymmetric character, which was similar to our HC group. However, the prevalence of hearing loss among the tinnitus patients in the studies makes it unclear whether the asymmetry in the left PAC reflects the abnormally spontaneous neural activity of the PAC. In a further PET study, Langguth et al. suggested that the asymmetry auditory cortical activity of tinnitus may not imply cortical hyperactivity and also have no correlation with tinnitus severity (Langguth et al., 2006). In our study, the mALFF values of the active cluster in the left HAC was found to be significantly correlated with clinical characteristics of tinnitus such as DTM, THI, and SDS (Figure 4). These results suggest that the activity cluster in the left HAC (BA22) might be involved in the changing baseline neural activity following tinnitus. Remarkably, the increased smALFF cluster was located in the left MTG (BA22) of TNH, the brain region links to the recognition of language and the selective attention to sound in human beings (Rinne et al., 2008).

Clinical therapy experience of tinnitus also had some pieces of evidence to support that the neural activity of HAC has a correlation with tinnitus severity. De Ridder et al. (2011) suggested that transcranial magnetic stimulation and extradural electrodes implanted over the HAC can help in tinnitus suppression. Plewnia et al. (2007) used PET to show attenuation of neural activity in the auditory cortical association area after transcranial magnetic stimulation in effectively treated patients. Emmert et al. (2017) found that tinnitus patients, who were performed neurofeedback treatment show weakened activity in the secondary auditory cortex, appeared lower relaxation index scores than pre-treatment, while no significant alteration in tinnitus loudness.

In similar RS-ALFF tinnitus research, Chen et al. found that increased spontaneous activity (measured with ALFF) in the right MTG (BA22), an auditory associated region, was correlated with tinnitus clinical characteristics, such as tinnitus duration and handicap score (Chen et al., 2014). The cause of the difference may be the tinnitus mechanism and the effect of the tinnitus side. From the study participants, all patients selected in our study had right ear tinnitus, and possible exposure factors were persistent non-invasive noise damage. As described in the previous literature, right-sided tinnitus caused by non-trauma noise exposure has a greater impact on speech recognition than left ear tinnitus and affects the severity of tinnitus symptoms (Tai and Husain, 2018). The location of abnormal activation in this study is just in the BA22 area on the left hemisphere, which have the function of recognition of language and the selective attention to sound in human beings (Rinne et al., 2008).

In animal studies, the mechanism of tinnitus following long-term exposure to non-traumatic noise might be the result of the cortical tonotopic map distortion in the PAC, which present decreased neural activity within the exposure frequency range and increased outside the exposure range. We did not find any difference in the PAC between TNH and HC, as we expected. Langguth et al. (2006) did not find a difference in the auditory cortices between subjects with tinnitus and controls using steady-state metabolism of PET evaluation. Chen et al. (2014) did not find different clusters in the PAC using the ALFF index. Only Shulman et al. (1995) presented hyperactivity (SPECT Tc^99^) in the PAC of tinnitus patients without hearing evaluation (Shulman et al., 1995). In our study, the null result of the PAC might be ascribed to the fact that the tonotopic mapping area of the PAC is too small to detect the difference for limitation of low spatial resolution RS-fMRI and small size of sample in our study.

In our study, decreased smALFF values are presented in the right IC. Various research studies with noise-induced tinnitus present increases in excitation and decreases in inhibition of the CAS, which be thought to stabilize the mean activity of neurons on long time scales (Schaette and McAlpine, 2011). Most of the tinnitus subjects in our study have environmental noise exposure and low activity of the right IC, which might be induced by adaptive homeostatic plasticity to environmental noise with wideband. Smits et al. (2007) reported a similar result showing that right ear tinnitus showed sound-evoked fMRI activation in the IC and lateralized toward the right IC. Landgrebe et al. (2009) also showed increased gray matter in the right IC of tinnitus, which means that that IC has more active neurons and increased neural activity. These are not consisted with our results. There is no correlation between the clinical characteristics of tinnitus and smALFF of the right IC. The IC activity might therefore be related to maintain sound perception (Schaette and McAlpine, 2011) rather than the clinical features associated with tinnitus.

In the present study, seed voxels were used to explore differences in brain-wide FC between the TNH and HC groups. When using a left HAC seed, we found increased FC with various brain regions including the bilateral supplementary motor area, bilateral STG, bilateral Heschl gyrus, bilateral insula, left amygdala, left hippocampus, right supramarginal gyrus, right cerebellum, and right inferior (Figure 5 and Table 3). When using the right IC seed, we did not find any difference in brain-wide FC. Interestingly, we also found that the smALFF values of the left HAC, but not the right IC, were significantly correlated with the clinical characteristics.

Although only the HAC in the central auditory nervous system shows an increased neural baseline activity and is related to clinical indicators of tinnitus, this change is not isolated to CAS. The activity cluster in HAC has stronger FC to auditory network-related brain regions (Mantini et al., 2007) include Heschl gyri (PAC) and bilateral anterior STG and right IC which means that the alteration of neural activity in HAC drives the aberrant functional linkages throughout the auditory network. Associative auditory cortices (BA22) have close relationship with Heschl gyri (PAC), which may cause the phantom sounds (Chen et al., 2014). The results suggest that the modulation of the resting-state auditory network neural activity in HAC affects the whole CAS, and previous studies found increased FC between the ipsilateral auditory cortex and the contralateral auditory cortex and STG (Burton et al., 2012; Kim et al., 2012; Maudoux et al., 2012a). The STG is thought to play a role not only in the perception of sound but also in cognition through a pathway consisting of the amygdala and prefrontal cortex (Bigler et al., 2007; Wild et al., 2012). The IC is a necessary relay in the ascending auditory pathway, a point at which virtually all lemniscal and extra-lemniscal ascending inputs converge (Aitkin and Phillips, 1984). In the sodium salicylate-induced tinnitus animal model, the secondary auditory cortex exhibited an increased firing rate (Eggermont and Kenmochi, 1998) as well as increased FC between the auditory cortex and the IC (Rinne et al., 2008; Chen et al., 2015). In our study, the HAC exhibited FC with the IC (Figures 3, 5), implying that the HAC is a major hub of tinnitus without hearing loss between the auditory network and various subnetworks. We hypothesize that the right IC receives a large descending projection from the HAC (BA22) and, in effect, modulates selective auditory attention to the tinnitus phantom sound perception.

The cerebellum is mainly thought to be associated with motor control and planning (Kelly and Strick, 2003; Konoike et al., 2012; Stoodley et al., 2012), and parts of this structure, such as supplementary motor area, lobule 4, the parafloccular lobe, and the vermis, have been found to contain links to the auditory system during tinnitus in animal models (Maess et al., 2001; Brozoski et al., 2007; Bauer et al., 2013). In particular, Chen et al. have suggested that the cerebellum (lobule 4) and parafloccular lobe form a gain control mechanism that compares the afferent input from the cochlea with descending signals from the auditory network (Chen et al., 2015). Consistent with this view, our results revealed abnormal activity in the HAC of tinnitus patients and an increase in the FC between this region and cerebellum lobules 4, 5, and 8.

The amygdala and hippocampus, which play roles in emotional responses and memory (fear, anxiety, and aggression), are members of the limbic system (Blair et al., 2001; Amunts et al., 2005). The extended object-attribute model study proposed that emotional aural events involve the auditory pathways (including the auditory cortex, IC, and thalamus) as well as the limbic system (Datta et al., 2005; McLachlan and Wilson, 2010). Interestingly, the increased FC between the HAC and IC, cerebellum lobules, amygdala, and hippocampus in the TNH group illustrates the network of the extended object-attribute model (Phelps, 2004). The neurophysiological model of tinnitus proposed by Jastreboff (1990) suggests an interplay between the auditory and limbic systems, which has been supported by various RS-fMRI studies that have also found evidence for this linkage in tinnitus patients (Kim et al., 2012; Maudoux et al., 2012a). Altogether, the results of our study provide further evidence that there exists an increased FC between the auditory cortex and the amygdala in tinnitus patients.

Some limitations of the current study should be considered when interpreting these findings. First, the study sample size is small and may be an issue when considering that small differences cannot always survive after multiple comparison statistical corrections and may induce erroneously active clusters. Therefore, we tried to maximize the differences between groups while remaining consistent across our patient populations by ensuring that they all had right ear tinnitus, a history of non-traumatic noise exposure, and no hearing loss. We also matched the patient groups according to age, sex, and education level, and excluded patients with various underlying diseases, such as metabolic diseases, long-term oral drug use, or emotional disorders. We attempted to select a stringent multiple comparison correction methods, the permutation test, which has been proved reliable (Rondinoni et al., 2013), to minimize the probability of statistical error. In the future, we will enlarge the sample size and patient groups to cases with right ear, left ear, and binaural tinnitus, as well as TNH and tinnitus with hearing loss, to further explore the mechanism of tinnitus. In addition, we cannot eliminate the substantial acoustic noise produced during the fMRI procedure, which might affect baseline neural activity (Amunts et al., 2005). We tried to reduce such interference by providing the patients with ear plugs and MRI noise-cancelling headphones, and by adjusting the pulse sequence before structural scanning (Paul et al., 2009). Finally, the abnormal FC between networks in the real world effects not only the resting-state brain but also the network dynamics (Di and Biswal, 2019). It is insufficient to observe the influence of tinnitus on the FC of the brain using only RS-fMRI and the LFF feature. How tinnitus impacts working efficiency, cognitive ability, memory, etc., needs to be further researched to determine an encompassing characterization of the disease.

## Conclusion

Tinnitus induced by no-trauma noise might be the result of abnormal spontaneous activity in the HAC and auditory pathway, which may subsequently cause plastic changes involving the increase in FC between the auditory network, cerebellum, and limbic system.

## Data Availability Statement

The datasets generated and analyzed during the present study are available from the corresponding author on reasonable request.

## Ethics Statement

The studies involving human participants were reviewed and approved by the Research Ethics Committee of Guangzhou Panyu Central Hospital and The First Affiliated Hospital of Jinan University, Guangzhou. The patients/participants provided their written informed consent to participate in this study.

## Author Contributions

TZ, Q-TY, and W-WC performed the design of the investigation and collected the subjects. Z-CL and W-WC contributed to the analysis of the resting-state fMRI data and wrote the manuscript. All authors have read and approved the manuscript.

## Conflict of Interest

The authors declare that the research was conducted in the absence of any commercial or financial relationships that could be construed as a potential conflict of interest.

## Footnotes

1 http://www.fil.ion.ucl.ac.uk/spm

2 www.fz-juelich.de/inm/inm-1/DE/Forschung/_docs/SPMAnatomy-Toolbox/SPMAnatomyToolbox_node.htm
